# Supplementary material for: Are carbon emissions trading and green financial instruments synergistic? -Comprehensive quantitative research based on content analysis
Source: PLoS One. 2024 Mar 7;19(3):e0298601. doi: 10.1371/journal.pone.0298601 (PMC10919601; doi:10.1371/journal.pone.0298601)
Supplement: S1 Data — (ZIP) [file pone.0298601.s001.zip › Data/地级市Z_F工作报告.zip!/┴╔─■/╙¬┐┌╩╨/└┤╫╘╣1⁄2╓┌║┼í╛┬φ┐╦╩2╛▌═°í┐_2009.docx]

各位代表：

　　现在，我代表市政府向大会报告工作，请予审议，并请市政协委员和列席会议的同志提出意见和建议。

　　一、2008年工作回顾

　　过去的一年，在省委、省政府和市委的正确领导下，在市人大、市政协的监督支持下，市政府坚持以邓小平理论和“三个代表”重要思想为指导，认真贯彻落实科学发展观，积极应对国际金融危机蔓延加剧和国内外复杂多变的经济形势，进一步解放思想，抢抓机遇，拼搏进取，通过全市人民的共同努力，国民经济和社会事业保持了又好又快的发展态势。预计全年实现地区生产总值703亿元，增长20.3%；固定资产投资543亿元，增长30%；社会消费品零售总额177亿元，增长23%；财政一般预算收入40亿元，增长37.7%；城市人均可支配收入1.445万元，增长19%；农民人均纯收入6950元，增长20%。

　　（一）推进沿海经济带建设，持续快速发展基础更加坚实

　　港口整体功能明显增强。全年完成基础设施建设投资25亿元，港口四期工程全部竣工投产，仙人岛港区等重点项目建设加速推进，集疏运能力显著提高。全年港口吞吐量完成1.5亿吨，增长22.9%；集装箱运量200万标箱，增长45.9%。B型物流保税中心获得国家批准，实现了港口发展的新突破。

　　沿海产业基地建设步伐加快。全年完成基础设施建设投资24.2亿元，华能营口热电厂、污水处理厂等一批重点工程顺利实施，产业和项目的承载能力显著增强。积极推进项目开发建设，新开工盼盼起重机、辽宁大族冠华等重点项目20项，完成投资60.7亿元。

　　开发区发展势头强劲。以“五区、三带、四廊、两园”开发建设为重点，积极拓展城市发展空间，“十分钟城市圈”发展框架和现代化、生态化的滨海城市雏形基本形成，综合竞争力不断提升。全年地区生产总值实现234亿元，各项主要经济指标增幅均在30%以上，开放先导区的引领作用进一步显现。

　　重点区域开发成效显著。高新区不断加快创新体系建设，开发、孵化和产业化功能不断完善。仙人岛能源化工区规划建设与项目开发同步推进，启动区基本具备项目入驻条件，65万立方米储运工程和80万立方米储罐项目全面竣工，50万吨重交沥青项目进展顺利。中小企业园起步势头良好，基础设施建设全面展开，8家企业开工建设。在沿海产业基地120平方公里重点发展区域基础上，又有四个区域、152平方公里被省政府列入沿海经济带重点支持区域。

　　（二）优化经济结构，综合经济实力显著增强

　　农业农村经济稳步增长。全年农业总产值实现105.4亿元，增长16.1%；农业增加值实现61.6亿元，增长7%。粮食生产喜获丰收，产量达到73.8万吨，蔬菜、水果、水产和畜牧业保持平稳较快发展。设施农业进一步强化，农机推广扎实推进，农业产业化水平不断提高。全市新建、扩建恒久果业、金马绢丝等规模以上项目23项，农产品加工业产值增长35%。大力实施品牌兴农战略，农业标准化进程全面提速，无公害、绿色、有机食品认证数量达到179个，大石桥市、盖州市通过了农业标准化暨“三品”认证整体推进县项目验收，被农业部列为北方水果无疫病控害区。14种农产品被确认为省名牌产品和省著名商标，其中“营口大米”获国家地理标志证明商标认证。农业基础设施不断完善，新增和改善灌溉面积7.8万亩，治理水土流失面积8.4万亩。县域经济保持良好发展态势，地区生产总值、财政一般预算收入等主要经济指标均超过全市平均水平。

　　工业经济整体实力显著提升。应对国际金融危机和国内经济形势新变化，市政府及时出台了促进中小企业平稳较快发展、搭建县域经济融资平台等一系列扶持政策，减小对工业经济运行的影响，项目园区化、产业集群化进程加快。全年新增规模以上企业160户，产值超亿元企业达到220户。规模以上工业总产值达到1330亿元，增长33%；增加值完成420亿元，增长27%。以冶金、石化、装备制造等产业为主导的新型工业体系框架正在形成，现代新型沿海工业城市特征日趋鲜明。科技创新能力不断增强，81个项目列入省重点创新项目计划，居全省首位。高新技术产业和循环经济快速发展，单位地区生产总值综合能耗下降4.5%，主要污染物排放控制在省计划以内。

　　服务业发展步伐加快。全年实现服务业增加值236亿元，增长17.2%。城乡市场繁荣稳定，城市商贸网点建设不断改善，农村市场快速发展，新兴业态不断壮大。现代物流业发展势头强劲，房地产业运行平稳，旅游业发展步伐加快，天沐温泉城、山海广场等一批重点项目相继开工建设。金融支持经济发展能力不断增强，新增贷款75亿元，不良贷款比率降至7.2%，省内首家村镇银行在我市投入运营。

　　（三）狠抓项目建设，对外开放呈现强劲势头

　　项目建设进展顺利。全年开工1000万元以上项目456项，完成投资418.9亿元，其中亿元以上项目127项。产业分布趋于合理，全市新建、续建服务业和社会事业项目40项，总投资55.6亿元。鞍钢鲅鱼圈新厂、中冶京诚（营口）中试基地、富士康科技园一期工程等重大项目建成投产，冶金、石化、装备制造等主导产业配套和产业延伸项目陆续跟进，现代临港产业格局初步形成。

　　招商引资工作成效显著。坚持以工业园区为载体，以项目建设为核心，转变招商方式，拓宽引资渠道，吸引内外资条件及项目承载能力明显改善和提升。全年新批外商投资企业98家，其中投资1000万美元以上企业39家；实际直接利用外资3.1亿美元，增长138%；内联引资实际到位220亿元，增长30%。

　　对外贸易持续快速发展。全年完成外贸进出口总额22.8亿美元，增长21.3%，其中出口完成16.8亿美元，增长24.2%。积极培育自主品牌，壮大外贸主体，新增自营进出口企业80家，自营企业出口增速高于三资企业24个百分点，158家企业进入国家产业损害预警系统，有效保护了企业贸易公平。

　　（四）实施民生工程，城乡群众生活条件明显改善

　　就业和再就业工作不断加强。以创建充分就业型城市为目标，认真开展就业援助工作，落实各项就业扶持政策，投入专项资金达1.3亿元。全市新增实名制就业8.2万人，普惠制培训5.8万人，农村剩余劳动力转移就业3.2万人。有劳动能力且有就业意愿的零就业家庭全部实现就业或双就业，城镇登记失业率控制在3%以下。

　　社会保障体系进一步完善。养老保障体系建设不断加强，企业养老保险新增5.4万人。医疗保障体系基本形成，城镇职工基本医疗保险新增7.4万人；城镇居民基本医疗保险全面启动，参保人数达到16万人；新型农村合作医疗参保人数达到110.4万人，参合率93%。住房保障体系建设加快推进，开工建设经济适用房40万平方米，完成廉租住房3万平方米，改造农村特困户危房800户，城乡特困群体住房条件明显改善。

　　扶贫帮困工作成效显著。新上扶贫开发项目36项，1.2万农村人口实现稳定脱贫，贫困人口下降12.3%。城乡救助水平不断提高，接收集中捐赠款物1165万元，救助城乡贫困群众5.4万户。医疗救助工作全面展开，城乡低保户、农村五保户和优抚对象看病难、看病贵问题有效缓解。建立城市特困群体冬季供暖补助机制，切实保障了“三无户”、低保户及特困户的冬季供暖。最低生活保障水平进一步提高，城市低保标准上调28.3%，实现应保尽保。积极援助四川灾区，先后派出卫生、消防等医疗救援队伍，为灾区捐款6700万元。

　　实事工程全面完成。坚持高质量建设、高标准管理，不断加大实事工程推进力度，年初确定的十件实事全部竣工。其中，改造街巷道路40条，比年初计划新增10条。实施焦化燃气综合利用，新增煤气用户1.2万户，比年初计划增加2000户。五大工程进展顺利，城市污水处理二期、大伙房水库输水配套等跨年度工程有序推进。实事征集过程中其它方面107件群众建议落实94件，占总数的87.9%。

　　城乡环境明显改观。城市发展总体规划不断完善，滨海带状城市规划基本完成。全年城市建设投资7.4亿元，比上年增长51%。城乡基础设施建设不断加强，石门水库供水、医疗废弃物处置中心等重点公益性项目进展顺利。公路交通建设步伐加快，在全省率先实现滨海大道全程竣工通车，率先实现“村村通油路”目标，按期完成营盖公路改造工程。电力对经济发展保障能力显著提升，全年投入电网建设改造资金11.4亿元，变电能力增长44.1%。加大城市绿化、亮化工作力度，全年新增绿地342公顷，市区安装亮化设施40处。不断强化城乡环境综合整治，市容市貌、环境卫生、市场管理、交通秩序明显改观，市区空气环境质量居全省前列，城市饮用水指标好于国家标准。

　　（五）发展各项社会事业，促进和谐稳定

　　教育事业优先发展。全年新增教育支出7359万元，增长13.3%。新建校舍3.5万平方米，改造农村中小学危险校舍21所，发放就学资助资金492.3万元，惠及贫困学生1.3万名。成功引进省实验学校、省实验中学等优质教育资源，校区建设分别在开发区、沿海产业基地开工。基础教育总体水平居全省前列，城乡义务教育均衡发展，非义务教育水平不断提升。

　　医疗卫生事业长足进步。全年投入资金925万元，改扩建乡镇卫生院19所，城乡公共卫生体系不断完善，应对突发公共卫生事件和急诊急救能力逐步提高。加强食品药品监管，市场安全稳定。人口计生服务能力不断增强，人口自然增长率1.31％。，人口结构日趋合理。

　　文化体育等各项事业繁荣发展。加速构建覆盖城乡的公共文化服务体系，加强社区和乡村文化设施建设。成功举办“欢乐中国行�D魅力营口”、“望儿山母亲节”等大型文化活动，城乡群众文化生活更加丰富多彩。广播电视网络和数字化进程不断加快，有线电视提前一年实现“村村通”。群众性体育活动蓬勃开展，投资320万元，新建健身路径86处，健身设施和场地明显改善，全民健身水平不断提高。市档案馆晋升为国家一级档案馆，双拥共建、国防教育、民兵预备役、国家安全、人民防空、民族宗教、外事侨务、妇女儿童、红十字、史志、老龄和残疾人等项事业取得长足进步。

　　和谐社会建设扎实推进。加强社会治安综合治理，处置突发事件和打击刑事犯罪的能力不断强化，圆满完成奥运安保任务。全年刑事案件发案率下降13.8%，人民群众安全感显著增强。认真排查调处各类矛盾纠纷，预防并妥善处理信访和影响社会稳定的突出问题，全市群众来信来访量下降20%。严格落实安全生产责任制，加强隐患排查和整改工作，安全生产总体保持平稳。

　　（六）加强政府自身建设，政务环境不断优化

　　“环境建设年”活动收效明显。“环境建设年”活动的开展，较好解决了非经济因素影响和制约经济社会发展的突出问题，全市上下的发展意识、服务意识和环境意识普遍增强。市政府行政审批服务中心投入运行，打造了优质、高效、便捷、规范的服务平台，发展环境显著改善。

　　民主法制建设进一步加强。认真贯彻落实市人大及其常委会的各项决议决定，自觉接受依法监督；坚持向市政协通报工作制度，自觉接受民主监督，认真办理人大代表议案、建议和政协提案、建议案。认真听取各民主党派、工商联、无党派人士和各人民团体的意见，民主决策渠道进一步拓宽。全年共办理人大代表建议223件、政协委员提案255件，办复率100%，办结率76%，满意和基本满意率100%。全面加强规范执法，政府行政行为步入法制化、规范化轨道。

　　政风建设不断强化。全面开展执法、廉政和效能监察，从严查处各类案件，坚决纠正损害群众利益的不正之风。扎实推进政务公开，公共管理和服务水平不断提高。切实增强政府工作人员的群众观念和公仆意识，公务员队伍的整体素质不断提高。全年市民投诉办结率达到91%以上，行政效能投诉案件下降24.7%。

　　各位代表，过去的一年是极不平凡的一年。如果说营口的发展已经引起了国内外的关注，那是沿海区位优势的实际彰显，是历届市领导班子工作成效的集中显现。过去一年所取得的工作成绩，是市委正确领导的结果，是市人大、市政协和社会各界监督支持的结果，是全市人民共同奋斗的结果。在此，我代表市政府向辛勤工作在各条战线的全市人民，向各级人大代表、政协委员、各民主党派、工商联、无党派人士、各人民团体，向离退休老领导、老同志，向驻营解放军、武警部队官兵和中省直单位，向所有关心、支持营口建设的同志们、朋友们表示衷心的感谢并致以崇高的敬意！

　　在肯定成绩的同时，我们也清醒地看到，全市经济和社会发展仍然存在一些不容忽视的问题：经济实力弱，县域经济发展不够平衡，财政紧张状况还没有从根本上得到缓解；农业产业化、农村城镇化水平不高，农民财产性收入和就业性收入比例偏低，持续增收的渠道不宽；自主创新能力不强，资源利用效率不高，粗放型增长方式尚未根本转变；服务业发展相对滞后，特别是现代服务业，与快速发展的社会需求、与第二产业发展要求、与沿海开放城市地位不相适应；制约中小企业发展的瓶颈问题没有得到有效解决，就业压力加大，劳动力结构性矛盾比较突出，社会保障能力有待进一步增强；个别政府部门和一些工作人员缺乏公仆意识、忧患意识，存在作风不实、效率不高、为政不廉的问题，政府自身建设需要进一步加强。对此，我们将采取有效措施，认真加以解决。

　　二、2009年主要任务

　　今年是中华人民共和国建国60周年，也是我市全面完成“十一五”规划的关键一年。当前，国际金融危机仍在蔓延加剧，国内外经济环境正在发生深刻变化，经济发展中的不稳定、不确定因素明显增加，我们将面临着更加严峻的考验和挑战。做好今年政府工作，对于战胜国际金融危机严重冲击，保持当前来之不易的好形势，维护改革发展稳定的大局，早日实现建设沿海经济强市的目标，意义十分重大。面对国际金融危机、经济下滑的严峻现实，面对省委、省政府对我们发展的殷切希望，面对全市人民对营口未来的美好企盼，在决定营口未来的这样一个关键历史时期，我们必须审时度势、坚定信心；必须发挥优势、挖掘潜力；必须攻坚克难、加快发展，把保增长作为头等大事，以昂扬向上的精神状态、求真务实的工作作风，开拓进取，扎实工作，开创营口经济社会发展的新局面。

　　全年经济社会发展的总体要求是：坚持以邓小平理论和“三个代表”重要思想为指导，深入学习实践科学发展观，全面贯彻落实党的十七大、十七届三中全会、中央经济工作会议和胡锦涛同志来辽宁考察时的重要讲话精神，按照市委十届九次全会的部署，以建设沿海经济强市为目标，继续实施以港兴市、工业强市战略，更加注重统筹城乡发展，完善产业布局，提升经济实力；更加注重改革开放和体制机制创新，增强发展动力，夯实发展基础；更加注重转变发展方式，优化经济结构，提高运行质量；更加注重改善民生，加快社会事业发展，促进社会和谐，保持经济社会又好又快发展，努力实现营口老工业基地在较高基点上的全面振兴。

　　全市经济社会发展的主要目标是：地区生产总值完成840亿元，增长18%；固定资产投资706亿元，增长30%；规模以上工业增加值550亿元，增长25%；社会消费品零售总额211亿元，增长19%；地方财政一般预算收入50亿元，增长25%；城市人均可支配收入1.619万元，增长12%；农民人均纯收入7990元，增长15%；城镇登记失业率控制在4%以下。

　　为实现上述目标，重点抓好以下六个方面工作：

　　（一）以港口发展建设为牵动，加速推进沿海经济带开发建设

　　港口是我们加快发展的战略资源、核心资源，建设沿海经济强市是政府工作始终不渝的奋斗目标。保持经济又好又快发展必须依托港口优势，加快构建开放型经济新格局。

　　加快港口发展建设。进一步加大对港口发展的支持力度，加快B型物流保税中心和仙人岛港区码头配套工程、A港池5#、6#码头等基础设施建设，全年完成固定资产投资25亿元。积极推进辽宁中部城市群出海产业大道营口段建设，不断完善陆港口岸系统，促进货源基地开发，建立起以港口为龙头的现代交通网络和物流体系。进一步加强港口服务，提高通关效率，优化服务环境，努力打造一流口岸。全年港口货物吞吐量完成1.7亿吨，集装箱运量240万标箱，为2010年实现港口吞吐量2亿吨奠定基础。

　　全力推进沿海经济带建设。沿海产业基地要超常规、跨越式发展，要突出项目建设核心，改善投资环境，提高服务质量；要加大项目建设力度，确保全年新开工项目80项以上，完成固定资产投资100亿元以上；要宽领域、多渠道招商引资，实际利用外资1.5亿美元以上；要加快完善城区功能和配套设施建设，全年开工商住、工业地产100万平方米以上。开发区要充分发挥港口优势，加快推进新型工业化、城市现代化和城乡一体化进程，加快引进重大项目、环保项目和高附加值项目。全年新开工亿元以上项目30项，地区生产总值等主要经济指标增幅确保在30%以上。高新区要坚持创新服务平台建设与项目开发引进紧密结合，全年新开工建设1000万元以上项目20项，主要经济指标增长30%以上。仙人岛能源化工区要做大园区规划、做优基础设施、做实园区项目，加快基础设施和项目建设，力争在重大石化项目建设上取得突破性进展，实现能源化工、港口物流和旅游度假三大产业协调发展。

　　全面提高对外开放水平。以“五点一线”沿海经济带重点发展区域和重点支持区域为载体，开展产业招商和专业招商，加强与世界500强和国内外大公司、大商社的合资合作。加强优势资源、基础设施、公共事业和流通服务业的项目包装推介，重点引进高端制造、高附加值的重大产业项目，推动利用外资与我市产业发展的战略对接，全年包装推介“四个一批”项目120项。鼓励企业股份制改造，引入战略投资者和合作伙伴，力争两户企业实现上市融资。认真组织好重大招商活动，全年实际直接利用外资3.7亿美元以上，内联引资260亿元以上。进一步调整出口商品结构，千方百计遏制矿制品和纺织服装产品出口下滑局面，积极开拓轻工、化工、机电产品和农产品国际市场。

　　（二）以优化产业结构为主线，进一步加快新型工业化进程

　　继续实施工业强市战略，坚持项目进园区、产业集约化发展方向，加快结构调整和产业升级，进一步壮大产业集群，提升工业经济整体实力。

　　加大项目建设力度。开展“项目建设年”活动，实施“5227工程”，即开工建设1000万元以上项目500项，亿元以上项目200项，全年新增规模以上工业企业200户，完成项目建设投资700亿元以上。坚持外引内联并举、大小项目并重，重点抓好鞍钢鲅鱼圈新厂、中冶京诚（营口）中试基地、富士康营口科技园、五矿宽厚板等一批续建项目建设，确保按计划推进；抓住国家投资拉动内需的有利机遇，积极推进机场、铁路、公路、港口及城际轨道交通等一批重大基础设施项目，最大限度争取国家政策资金支持；抓紧做好一批重大项目的筛选论证、包装推介，建立项目动态生成机制，充实项目储备。建立完善项目定期考核制度，加强对签约项目和在建项目的跟踪服务，提高项目履约率、开工率和投产率，努力实现项目建设的新突破。

　　加快推进工业园区建设。采取多种形式广开融资渠道，加速园区开发建设，优化资源配置，强化要素支撑，提升园区承载力和配套服务功能。以“一港、三区、两基地”为支撑，统筹推进各园区开发建设，重点抓好中小企业园、开发区临港和滨海工业区、大石桥南楼和环城开发区及盖州北海新城等重点园区建设。加快项目园区化步伐，支持各市（县）区跨区域摆放项目，鼓励城区企业向园区转移，控制园区外新上工业项目，促进工业集中布局、产业集群发展、资源集约利用，努力把园区建设成产业集聚的新高地、提升实力的增长极。

　　培育壮大产业集群。进一步加大对重点骨干企业的扶持保障力度，支持优势企业抓住经济调整的有利时机，兼并、收购、重组国内外停产、半停产企业，实现低成本扩张。进一步加快产业集群化进程，以推进冶金、石化、装备制造等六大产业集群化发展为重点，围绕加快冶金产业发展，进一步完善产品配套，拉长产业链条，加快建设新兴精品钢材生产基地；围绕石油化工和精细化工产业发展，着力推进仙人岛能源化工区建设，加强与中海油等大企业合作，努力打造新兴能源化工产业基地；围绕大型重装备制造、船舶修造等系列生产，加快推进中小船舶制造、汽保设备等行业发展，壮大新兴装备制造产业基地；围绕镁合金、镁化工产品开发和资源综合利用，不断提高镁制品深加工比重，做优做强镁质材料产业基地；围绕纺织服装业稳定发展，促进上下游产品的内在链接，提高行业创效能力，加快建设领先辽宁的纺织服装生产基地；围绕壮大新型建材产业，加大培植力度，提升行业集群化水平，努力建设新型建材基地。全年六大产业集群产值达到1360亿元以上，全市规模以上工业总产值达到1750亿元以上。

　　扶持中小企业发展。要突出重点，加大政策扶持力度，减轻企业负担，帮助中小企业渡过难关。进一步淘汰落后生产能力，形成一批科技含量高、市场前景好、利润空间大的中小企业群体。努力拓展中小企业融资渠道，组建3家中小企业贷款公司。进一步加强中小企业担保体系建设，鼓励各商业银行加大信贷投放力度，为中小企业发展提供资金支持。充分发挥中小企业创业孵化基地作用，引导企业加速产业结构升级，提高抵御市场风险能力。继续推进中小企业服务平台建设，进一步完善“三级”服务体系，加强创业指导，降低创业成本，建立和完善中小企业生成机制。

　　认真做好节能减排。加快科技进步与创新步伐，全年实施投资500万元以上技术改造项目120项，工业企业重点技术创新项目130项，促进产业升级、产品更新换代。严格执行项目环境影响评价和能耗评估审批制度，完善环境、用能准入机制，控制高耗能、高污染的小化工、小钢铁等项目，提高项目准入标准。强化重点行业、重点企业的环境与能源审批和节能减排考核，全年实施工业资源节约与综合利用项目30项。以清洁生产和综合利用为重点，大力发展循环经济，促进可持续发展，抓好城市污水处理厂、垃圾无害化处理厂和电厂脱硫等重点工程建设。全年单位地区生产总值综合能耗下降4.5%以上，化学需氧量、二氧化硫排放量分别减少3.2%和2.4%。

　　（三）以发展县域经济为载体，积极推进社会主义新农村建设

　　进一步深化农村改革，大力发展县域经济，推进农业区域化、规模化经营，产业化、标准化发展，加快城乡一体化进程，促进农业增效、农民增收。

　　加快县域经济发展。坚持以园区建设为着力点，以项目为载体，大力培育支柱产业和特色产业，优化区域经济布局，做大县域经济总量。全面推进城镇化和城乡一体化进程，发展具有地方特色和优势明显的中心城镇，增强城镇的辐射能力和聚集效应。统筹区域发展规划和产业布局，强化开发区的辐射带动作用，推进开发区和盖州市资源共享，优势互补，一体化发展；推进大石桥市、老边区多元合作，互利共赢，协调发展。全力实施县域经济三年倍增计划，用好用足各项政策，建立以工促农、以城带乡的长效机制，全年县域经济生产总值、固定资产投资、财政一般预算收入的增幅确保达到25%、35%和30%以上。

　　强化农业基础设施建设。加强水土流失治理、河道生态改造和病险水库加固工程建设，全年新增灌溉面积1.2万亩，治理水土流失面积8万亩。推进农村生态建设，全年人工造林4万亩，义务植树600万株。建立完善农村气象灾害预警系统，全面推进农业政策性保险，切实做好防灾减灾工作。坚持多元化投入，积极筹措支农资金，扎实推进试点村建设，着力改善农村生产生活条件，全面提高新农村建设水平。

　　发展现代农业。按照高产、优质、高效、生态、安全的要求，进一步调整和优化农业结构，全年粮食产量稳定在70万吨，蔬菜、水果、水产品、畜产品产量稳定增长。积极促进规模化发展，全年新建设施农业种植小区521个，畜牧小区112个，渔业小区17个。大力推进产业化经营，新建、续建投资500万元以上龙头项目20项，农产品加工业产值增长30%以上；订单农业新增10万亩。加强农村社会化服务体系建设，新建专业合作社100个。加大农业机械化推广力度，增加农机具补贴，综合机械化水平达到57%以上。不断加强标准化建设，新增“三品”认定面积10万亩，新增认证数量60个。继续实施“品牌兴农”战略，全年新增市级品牌30个，省级品牌10个，国家级品牌3个。

　　创造农村发展的良好环境。稳定并完善农村基本经营制度，在保持土地承包关系持续稳定的基础上，积极探索农村集体土地流转的新方式和多种形式的适度规模经营；完善农业支持保护制度，保证各级财政对农业投入增长幅度高于经常性收入增长幅度；建立现代农村金融体系，深化农村信用社改革，促进村镇银行和小额贷款公司发展，建立多元化的农业投入机制；建立促进城乡经济社会发展一体化制度，促进公共资源在城乡之间均衡配置、生产要素在城乡之间有效流动；健全农村民主管理制度，推进村民自治制度化、规范化、程序化。

　　（四）以滨海带状城市规划实施为契机，全面提升城乡建设管理水平

　　滨海带状城市是建设“大营口都市区”的前提和基础。按照“滨海、带状、组团、生态”的总体规划要求，加快滨海带状城市规划实施步伐，努力把营口建设成为产业人口集聚能力强、综合承载能力提升快的大型现代化海滨城市。

　　坚持高起点规划高标准建设。认真做好《营口市城市总体规划》和《营口市土地利用总体规划》修编，加快“大营口都市区”建设进程，着力构建滨海带状城市新格局。进一步做好沿海产业基地及各类工业园区修建性详细规划，完善新农村建设规划，保证规划的科学性和严肃性。加快城市道路、广场、供暖等基础设施建设，继续实施绿化、美化、亮化工程，积极推进大伙房水库输水配套、渡口老街改造等一批续建工程建设，进一步完善城市功能，提升城市整体形象。

　　全面加强城乡管理。加大城市综合执法力度，集中整治乱贴乱画、乱停乱放、乱搭乱建等违法违章行为，努力创造优美、整洁的市容环境。进一步强化交通管理，整顿交通秩序，规范客货运输市场管理。加强市政设施管理和维护，大力推进城市集中供热和物业管理、环卫保洁等规范化管理，不断提高城市公共服务水平。深化社区管理体制改革，逐步建立承担公共服务职能的社区工作新机制。大力推进城市管理向镇村延伸，搞好农村集贸市场、街巷道路和城乡接合部等的综合整治，使乡村环境有较大改观。

　　加强环境保护和生态建设。严格执行“环保第一审批”和“一票否决”制度，进一步优化生态环境。继续抓好生态湿地保护，加大对大辽河、大清河、碧流河等流域污染治理力度，确保水质达到水环境功能区标准。搞好熊岳河的综合整治，做到统筹规划，两岸同步开发。严格落实对山体、森林、岸线及海域等生态环境资源的保护措施，坚决禁止盲目开发，切实保护好营口的青山绿水、碧海蓝天。

　　加快发展服务业。坚持把扩大消费需求作为拉动经济增长的着力点，改善消费预期，优化消费结构，增强消费拉动能力。发展壮大现代物流业，大力推进8个专业物流基地和7个区域性物流配送中心建设，构建覆盖东北的物流网络。巩固提升商贸服务业，做大做强社区服务。培育和引进经营规模大、辐射能力强的商贸中心和专业市场，创区域特色和地区品牌，提高现代流通业规模化和集约化水平。进一步深化金融生态市建设，积极引进金融机构，加大金融担保体系建设，建立金融服务平台，推动金融业加快发展。加速发展房地产业，进一步优化房地产开发环境和开发结构，全年房地产业增加值达到19亿元以上。大力发展旅游业，以温泉资源开发为牵动，整合旅游资源，打造营口温泉城市品牌，形成特色鲜明的旅游产业。全年第三产业增加值力争实现与地区生产总值同步增长。

　　（五）以保障和改善民生为重点，扎实推进和谐社会建设

　　坚持以人为本，倾力改善民生，认真解决好人民群众最关心、最直接、最现实的利益问题，让人民群众共享振兴发展的成果是执政为民的根本要求。

　　努力做好就业和社会保障工作。以创建“充分就业城市”为目标，针对就业形势新变化，以城镇下岗职工、返乡农民工和特困群众为重点，进一步加大就业再就业援助力度，全年实现城镇实名制就业6万人，农村剩余劳动力实名制转移就业1.5万人，确保零就业家庭动态为零。以创业带动就业，加强创业培训，健全创业培训体系。全年实名制培训城镇下岗失业人员1万人，实名制培训进城务工农民1.4万人。高度重视大中专毕业生就业问题，采取积极措施，吸引优秀人才回乡创业。继续扩大城镇企业职工养老保险覆盖范围，年内参保人数达到58.5万人，其中在职职工参保人数达到42万人，确保企业离退休人员养老金按时足额发放。全力做好城镇居民医保扩面工作，城镇居民参保率达到80%。严格执行最低生活保障制度，建立有进有出的动态管理机制。加强扶贫开发，全年减少低收入贫困人口1.5万人。建立健全覆盖城乡特困群众的社会救助体系，切实改善特困群体生活。

　　全面发展社会各项事业。坚持教育优先发展，进一步完善义务教育的政府保障机制，使公共财政向教育倾斜、教育资源向农村倾斜，促进城乡教育均衡发展。抓好省实验中学和省实验学校分校建设，力争实现夏季招生。加快推进营口大学园建设，为创建本科大学奠定基础。大力推行工学结合、校企合作的人才培养模式，不断强化职业教育。坚持医疗卫生的公益性质，积极探索医疗卫生改革，优化公共卫生资源配置，推进社区和乡镇卫生院标准化建设，改善城乡医疗条件。加强公共卫生体系建设，提高疾病预防控制和应对突发公共卫生事件能力。健全完善公共文化服务体系，繁荣文化事业，发展文化产业，打造特色文化。积极推进数字电视转换工作，促进广播电视事业发展。认真贯彻计划生育基本国策，人口自然增长率控制在1.93％。以下，出生人口综合性别比控制在正常值范围。广泛开展群众性体育活动，努力提高竞技体育水平。做好双拥共建工作，加强国防教育和民兵预备役、国家安全、人民防空建设，努力提高妇女儿童、老龄、民族宗教、外事侨务、档案、史志等各项工作水平。

　　实施“五大惠民工程”办好“十件实事”。 “五大惠民工程”是：（1）城乡饮水安全工程。以水源地保护、大清河流域治理、农村饮水设施建设为重点，用3年时间彻底解决城乡居民饮水安全问题。（2）温暖清洁工程。为进一步提高居民供暖质量和城市环境质量，力争用4年时间，实现主城区2�D3个热源厂、市（县）区1个热源厂集中供热，并分期分批对供热管网进行改造。（3）城市“退二进三”工程。坚持政府主导，市场运作，用4年时间，将坐落在城区内重点部位的工业企业迁至工业园区，为人民群众营造良好的生活环境。（4）城中村改造工程。统筹规划，分期实施，用4年时间，对老边区欢心甸村、太和庄村等城中村进行改造，改善人居环境，加快城市化进程。（5）民兴河改造工程。按照景观河、生态河的标准改造民兴河，使其成为连接新老城区的绿化带、景观带；实施大辽河河口堤防整治，完善城市防洪体系。全年要重点为城乡群众办好“十件实事”：（1）改造城区道路25条、庄林路6.4公里；（2）改造旧小区8个、旧住宅楼41栋；（3）改造农村厕所2500座；（4）新建回迁楼20万平方米、廉租房3万平方米；（5）新建农村生态能源10处、沼气池3000户；（6）改造市区8所中学和乡镇10所中小学操场；（7）改扩建市中医院；（8）组建市农产品检验检测中心；（9）新增农机具1200台套；（10）组建7所农村区域性中心敬老院。

　　切实维护社会稳定。深入开展“五五”普法宣传教育活动，加强平安营口建设。继续抓好社会治安综合治理，建立预防、管理和打击相结合的治安防控体系，进一步增强人民群众的安全感。全面开展“解难题促和谐”实践活动，充分发挥信访、市民投诉中心、民心网和基层司法所的职能作用，畅通群众诉求渠道，严格落实信访工作责任制，有效化解各类矛盾，促进社会和谐。认真落实安全生产责任制，强化行政领导工作责任和企业主体责任，加强重点领域、重点行业安全隐患的排查和整治，高度重视食品、药品、交通和消防安全，严防涉及人民群众生命财产安全的重特大事故发生。

　　（六）以服务群众为宗旨，努力建设人民满意政府

　　建设执政为民、奋发有为、务实创新、廉洁高效的人民政府，既是经济社会发展的客观要求，也是政府自身建设的不懈追求。

　　坚持解放思想、开拓进取。进一步树立尊重实践、尊重规律，勇于创新、锐意进取的思想观念，使政府工作切实体现不畏艰险、直面挑战、敢为人先的精神风貌。不断创新思维，增强大局意识、发展意识和责任意识，坚决打破一切束缚发展的条条框框，进一步转变政府职能，下放管理权限，健全监督机制，形成充满活力、富有效率、更加活跃、有利于科学发展的体制机制，激发全市上下大发展、快发展的浓厚热情，让创新思想充分活跃起来，让创业积极性充分调动起来，让创造潜能充分释放出来，形成加快发展的强大合力。

　　坚持依法行政、民主决策。认真贯彻落实人大及其常委会的各项决议决定，加强与人民政协的政治协商，进一步强化人大代表议案、建议和政协委员提案、建议案办理落实力度，密切同各民主党派、工商联、无党派人士和各人民团体的联系。继续完善社情民意反映制度、社会听证制度和专家咨询制度，市政府召开涉及全市性重大决策和民生问题会议，邀请人大代表、政协委员和群众代表列席，促进决策民主化、科学化。增强政府工作的公开度、透明度，多渠道向社会发布政务信息。加强行政执法队伍建设，建立权责明确、行为规范、监督有效、保障有力的执法体系。认真落实行政执法责任制，严格规范行政行为，提高依法行政的能力和水平，做到有权必有责、用权受监督、侵权须赔偿、违法必追究。

　　坚持以人为本、勤政为民。认真落实事关群众利益的政策要求，在特殊困难时期，多为企业着想、为群众着想、为大局着想。及时解决群众反映的实际问题，坚持不懈为民解忧、为民谋利、为民造福，真正做到重大决策依民而定，工作措施应民而出，安危冷暖唯民而系，多做群众急需之事，多做百姓受益之事，多做为长远发展打基础之事，使政府的各项工作真正体现群众意愿，符合群众利益，满足群众需求。

　　坚持求真务实、真抓实干。结合开展学习实践科学发展观活动，巩固和发展“环境建设年”成果，为企业创造更加宽松适宜的发展环境。加强决策监督，完善政府部门之间的沟通、协调、配合机制，增强政府执行力，确保政令畅通。大力倡导不事张扬、埋头苦干的作风，坚决反对形式主义和官僚主义，不搞劳民伤财的“形象工程”和脱离实际的“政绩工程”。坚持谋实策、重实效，努力建设诚信政府。

　　坚持勤俭节约、艰苦奋斗。大兴艰苦奋斗之风，牢固树立过紧日子的思想，坚持厉行节约、勤俭办事，加强行政机关和事业单位经费、资金管理，反对奢侈浪费。加大财政资金监管力度，优化财政支出结构，着力控制和压缩一般性行政支出，降低行政成本，将有限的资金和财力用在促进发展和改善民生上，努力建设节约型政府。

　　坚持从严治政、廉洁从政。认真执行廉政建设责任制，严格落实“一岗双责”，切实把廉政建设贯穿政府工作的全过程。坚持标本兼治，充分发挥监察、审计等监督机关的作用，以工程建设、土地出让、政府采购、资源开发、社会保障等领域为重点，强化对权力运行的制约和监督，严肃惩处违纪违法行为。加强制度建设，提高源头防范能力，构建反腐倡廉的长效机制，努力建设人民满意的政府。

　　各位代表，营口的发展正处在继往开来的重要战略机遇期。立足新起点，应对新挑战，推进大发展，是时代赋予我们的光荣使命。让我们紧密团结在以胡锦涛同志为总书记的党中央周围，在省委、省政府和市委的正确领导下，紧紧依靠全市人民的智慧和力量，以更加开放的发展理念、更加执着的创新精神、更加扎实的工作作风，抢抓机遇，迎难而上，万众一心，奋力拼搏，为实现经济社会又好又快发展的奋斗目标，开创营口更加美好的未来而努力奋斗！
